# Small Interfering Ribonucleic Acid as Lipid-Lowering Therapy: Inclisiran in Focus

**DOI:** 10.3390/ijms24066012

**Published:** 2023-03-22

**Authors:** Jelena Rakocevic, Milan Dobric, Rada Vucic, Matija Furtula, Ivan Zaletel, Katarina Milutinovic, Ana Ilijevski, Milica Labudovic Borovic, Miloje Tomasevic, Milos Bajcetic

**Affiliations:** 1 Institute of Histology and Embryology “Aleksandar Đ. Kostić”, Faculty of Medicine, University of Belgrade, 11000 Belgrade, Serbia; jelena.rakocevic@med.bg.ac.rs (J.R.);; 2 Institute for Cardiovascular Diseases “Dedinje”, 11000 Belgrade, Serbia; 3 Faculty of Medicine, University of Belgrade, 11000 Belgrade, Serbia; 4 Cardiology Clinic, University Clinical Center Kragujevac, 34000 Kragujevac, Serbia; 5 Department of Internal Medicine, Faculty of Medical Sciences, University of Kragujevac, 34000 Kragujevac, Serbia; 6 Cardiology Clinic, University Clinical Center of Serbia, 11000 Belgrade, Serbia

**Keywords:** siRNA, small interfering RNA, inclisiran, LDL cholesterol, PCSK9

## Abstract

The PCSK9 (Proprotein Convertase Subtilisin/Kexin type 9) enzyme interferes with the metabolism of low-density lipoprotein (LDL) cholesterol. Inhibition of PCSK9 results in lower LDL cholesterol levels, which can be achieved by different molecular pathways. Monoclonal antibodies targeting circulating PCSK9 have shown strong and persistent effects on lowering the LDL cholesterol level, while reducing the risk of future cardiovascular events. However, this therapy requires once- or twice-monthly administration in the form of subcutaneous injection. This dosing regimen might impact the therapy adherence in cardiovascular patients who often require multiple drugs with different dosing intervals. Small interfering ribonucleic acid (siRNA) represents a promising therapy approach for patients with elevated LDL cholesterol level despite optimized background statin therapy. Inclisiran is a synthesized siRNA which inhibits PCSK9 synthesis in the liver and provides sustained and durable lowering of LDL cholesterol with twice-yearly application and a good tolerability profile. Herein, we present an overview of the current available data and critical review of the major clinical trials which assessed safety and efficacy of inclisiran in different groups of patients with elevated LDL cholesterol level.

## 1. Introduction

An elevated level of low-density lipoprotein (LDL) cholesterol represents a major, yet modifiable risk factor for the development of atherosclerotic cardiovascular disease (ASCVD). Since the longstanding elevation in LDL cholesterol demonstrates a cumulative effect on ASCVD risk, early diagnosis followed by adequate diet, lifestyle changes, and lipid-lowering therapy could substantially reduce the risk of future cardiovascular events [1]. Statins remain the first-line therapy for controlling LDL cholesterol levels. In cases where the LDL cholesterol goal is not achieved, a statin combination with ezetimibe should be considered [2]. However, this type of lipid-lowering therapy requires daily administration and, therefore, can lower therapy adherence. On the other hand, statins exert a variable therapeutic response; together with lower compliance, this might be the reason why certain patients do not reach guideline-recommended LDL cholesterol targets [3].

Inhibition of PCSK9 (Proprotein Convertase Subtilisin/Kexin type 9) by monoclonal antibodies, such as evolocumab or alirocumab, provides an additional approach for LDL cholesterol reduction. PCSK9 inhibitors have proven their safety and efficacy in lowering LDL cholesterol levels by about 50–60% compared to placebo [4,5]. Adding a PCSK9 inhibitor to maximally tolerated statin therapy resulted in lowering the risk of future adverse cardiovascular events by 15% [6,7]. However, monoclonal antibodies are expensive drugs and require subcutaneous administration every 2 weeks or once a month, which can add to lower therapy adherence [8].

A novel, molecular-based approach in lowering LDL cholesterol level is achieved by the inhibition of PCSK9 synthesis. Inclisiran represents small interfering ribonucleic acid (siRNA) which blocks PCSK9 synthesis in the liver, reducing LDL cholesterol in a comparable manner as PCSK9 monoclonal antibodies [9]. Three phase 3 studies have proven the safety and efficacy of inclisiran in patients with familial hypercholesterolemia, ASCVD, and ASCVD risk equivalents [10,11]. Promising and positive study endpoints resulted in approval of inclisiran along with diet and maximally tolerated statin therapy for adults with heterozygous familial hypercholesterolemia or clinical ASCVD who require additional therapy for lowering LDL cholesterol. Twice yearly administration is an additional benefit of RNA-based lipid-lowering therapy, which can profoundly increase therapy adherence in cardiovascular patients. Ongoing VICTORION-2 Prevent [12] and ORION-4 [13] trials will show whether such a potent lipid-lowering effect of inclisiran translates into lower cardiovascular risk.

Here, we present the results and a critical review of the major clinical trials which assessed the safety and efficacy of inclisiran in different groups of patients with elevated LDL cholesterol level.

## 2. siRNA-Based Therapy: How Does It Work?

**Figure 1:**
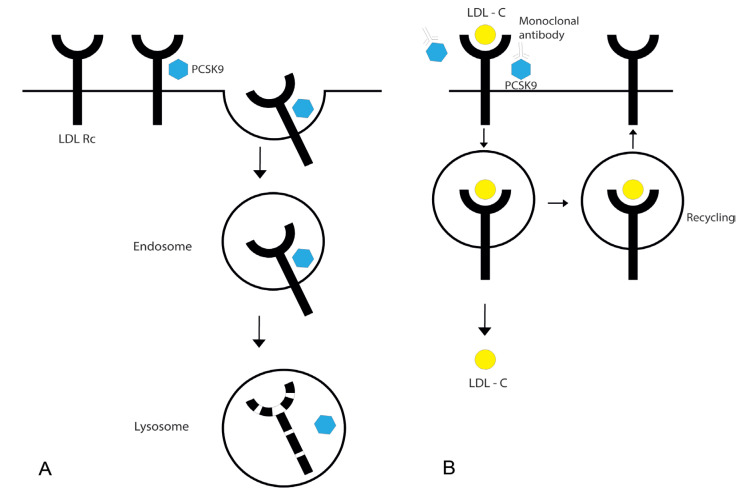
Effects of PCSK9 and PCSK9 monoclonal antibodies on LDL receptors. PCSK9 enzyme is secreted by hepatocytes, while LDL receptors are expressed on the surface of these cells. Upon secretion, PCSK9 binds to the extracellular region of LDL receptor. PCSK9/LDL receptor complex undergoes endocytosis and fusion with lysosomes, which results in their degradation (**A**). PCSK9 monoclonal antibodies bind circulating PCSK9 and inhibit its effect. As a result, LDL receptors remain longer on the surface of hepatocytes and undergo recycling after the internalization of LDL cholesterol (**B**). LDL Rc—LDL receptor; PCSK9—Proprotein Convertase Subtilisin/Kexin type 9; LDL-C—LDL cholesterol.

PCSK9 enzyme is mainly produced in the liver and is engaged in the degradation of LDL receptors. Upon secretion, PCSK9 binds to the LDL receptors on the surface of hepatocytes. The PCSK9/LDL receptor complex then undergoes endocytosis and fusion with lysosomes, which results in their degradation [14] (Figure 1A). Therefore, the PCSK9 enzyme indirectly supports the elevated values of LDL cholesterol in circulation. Synthesized monoclonal antibodies, such as evolocumab and alirocumab, bind circulating PCSK9 and inhibit its effect. As a result, LDL receptors remain longer on the surface of the hepatocytes, removing the excess LDL cholesterol from circulation [14] (Figure 1B).

In addition to the strong lipid-lowering effect, PCSK9 monoclonal antibodies have proven cardioprotective effects in patients with elevated LDL cholesterol levels. Specifically, in patients with ASCVD and LDL cholesterol levels of ≥1.8 mmol/L despite statin therapy, evolocumab reduced the risk of major adverse cardiac events (MACE) by 15% compared to placebo [6]. Long-term administration of alirocumab in patients with recent acute coronary syndrome and elevated LDL cholesterol levels led to 15% risk reduction in MACE compared to placebo [7].

However, PCSK9 inhibitors need to be administered by subcutaneous injection once or twice per month. Since cardiovascular patients with dyslipidemia often require multiple drugs with different dosing regimens, adding another drug even once or twice monthly might increase medication burden and lower therapy adherence [15]. On the other hand, poor adherence is strongly associated with adverse cardiovascular outcomes [3], which is why new therapeutic options for reducing LDL cholesterol are being developed.

RNA interference is a natural and highly conserved process occurring in all mammalian cells. This process is important for gene silencing and is mediated by noncoding, double-stranded siRNAs. Binding siRNA to a specific intracellular complex (RNA-induced silencing complex, RISC) enables catalytic cleavage of complementary messenger RNA (mRNA), inhibiting its translation into polypeptide chain [16,17].

**Figure 2:**
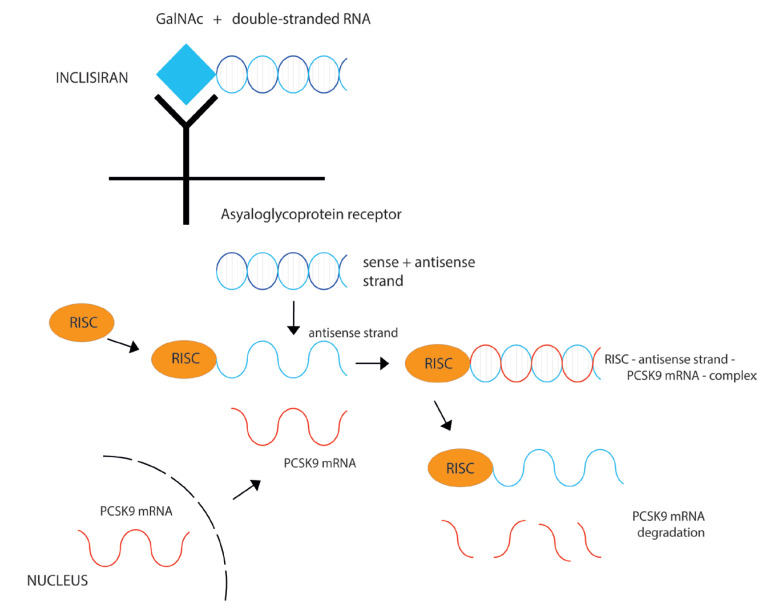
Mechanism of LDL cholesterol-lowering effect of inclisiran. Inclisiran consists of double-stranded RNA conjugated with N-acetylgalactosamine carbohydrates (GalNAc). GalNAc modification of inclisiran allows specific binding to asialoglycoprotein receptors present on hepatocytes. After its uptake by the liver cells, inclisiran binds RISC, as well as mRNA encoding PCSK9. In that way, the inclisiran–RISC complex degrades mRNA and prevents PCSK9 synthesis. RISC—RNA induced silencing complex; mRNA—messenger RNA; PCSK9—Proprotein Convertase Subtilisin/Kexin type 9.

Inclisiran is a synthesized siRNA that inhibits translation of mRNA into PCSK9 enzyme (Figure 2). Specifically, inclisiran consists of two nucleotide strands (sense and antisense strand) conjugated with N-acetylgalactosamine carbohydrates (GalNAc). GalNAc modification of inclisiran allows its specific binding to asialoglycoprotein receptors abundantly present on the liver cells, enabling rapid uptake of inclisiran by hepatocytes [18]. Peak plasma levels of inclisiran are achieved after 4 h of administration, while inclisiran is no longer detected in plasma after 24–48 h [16,19]. Inclisiran has a short plasma half-life due to its rapid and specific uptake by hepatocytes. However, there is no need for inclisiran dose adjustments in patients with mild to moderate hepatic dysfunction, since inclisiran was undetectable in plasma by 48 h, regardless of hepatic function [20].

After its uptake by the liver cells, the antisense strand of inclisiran binds RISC, as well as mRNA encoding PCSK9. In that way, the inclisiran–RISC complex degrades mRNA and prevents PCSK9 synthesis [18]. Despite its short half-life in plasma, inclisiran loaded into RISC complex can degrade multiple PCSK9 mRNAs, which might explain the sustained effect of inclisiran (up to 6 months after the administration) (Figure 2). The final outcome of inclisiran administration is the same as with PCSK9 inhibitors; LDL cholesterol receptors remain longer on the surface of hepatocytes, promoting LDL cholesterol clearance from the blood. The main difference is that inclisiran has a prolonged effect and requires administration every 6 months, compared to more frequent administration of PCSK9 inhibitors. The safety profile of PCSK9 inhibitors and inclisiran appears comparable, with injection site reactions being the most common adverse event due to subcutaneous administration.

siRNA-based drugs are already being used in the treatment of certain rare diseases, such as transthyretin amyloidosis and porphyria [21,22]. However, approval of inclisiran as a lipid-lowering agent widens the therapeutic potential of siRNA and introduces nucleic acids into the therapy of more common diseases.

## 3. Effects of Inclisiran in Healthy Population: Phase 1 Trial

The safety and pharmacodynamics of inclisiran were tested in 69 apparently healthy volunteers with no prior cardiovascular disease, cerebrovascular disease, or diabetes mellitus. Included participants had LDL cholesterol level of 2.6 mmol/L or higher despite background statin therapy [23] (Table 1).

This randomized, single-blind, placebo-controlled study tested inclisiran in a single-dose regimen and a multiple-dose regimen. Firstly, patients were randomized into six groups and received a single subcutaneous injection of inclisiran (25, 100, 300, 500, or 800 mg) or matching placebo. Secondly, the multiple-dose study phase randomized participants into six groups: (1) inclisiran 125 mg weekly for four doses, (2) inclisiran 250 mg every other week for two doses, and (3) inclisiran 300 or 500 mg monthly for two doses, with or without concurrent statin therapy [23].

Since this was a phase 1 trial, the results primarily focused on safety and pharmacodynamic variables as a surrogate of efficacy. All adverse events were mild or moderate in severity, with no drug discontinuation due to the adverse events. Furthermore, no serious adverse events were reported. Patients receiving a single dose of inclisiran most commonly reported cough, musculoskeletal pain, and nasopharyngitis (11% in each group). In the multiple-dose study arm the most common adverse events were headache (18%), back pain (15%), diarrhea (15%), and nasopharyngitis (12%). Injection-site reactions were not reported [23].

As for pharmacodynamic profile, PCSK9 level and LDL cholesterol level were measured on day 84 and compared with baseline values. Single administration of inclisiran (dose 300 mg or more), as well as all multiple dosing regimens, significantly reduced PCSK9 and LDL cholesterol levels by the day 84. A single dose of inclisiran of at least 100 mg reduced LDL cholesterol level by a least-squares mean of 36.7% to 50.6%. Inclisiran showed sustained effect after 6 months of administration, but only in groups who received this siRNA at the dose of 300 mg or more. The multiple-dose regimen showed even stronger effects on PCSK9 and LDL cholesterol levels. The reduction in LDL-cholesterol ranged from 45.1% to 59.7%, with the greatest reduction observed with 300 mg of inclisiran once monthly for 2 months [23].

With little variation in LDL cholesterol-lowering effects during the 180 days after the first dose, preliminary results encouraged less frequent inclisiran application than with PCSK9 monoclonal antibodies (e.g., once in 3 months or even once in 6 months). These assumptions need confirmation on a larger population of patients.

## 4. High Cardiovascular Risk and Elevated LDL Cholesterol: ORION-1 Trial

ORION-1 was a double-blind, placebo-controlled phase 2 trial which demonstrated the dose-dependent effect of inclisiran on PCSK9 and LDL cholesterol levels in patients with high cardiovascular risk and elevated LDL cholesterol levels [24]. This was a dose-finding study, but ORION-1 also explored dosing intervals of inclisiran in patients with elevated LDL cholesterol level despite maximally tolerated statin therapy. The threshold for LDL cholesterol levels was different, depending on whether patients had known ASCVD (LDL cholesterol >1.8 mmol/L) or no known ASCVD (>2.6 mmol/L).

Patients were randomized into eight study groups; four groups received a single dose of inclisiran (200, 300, or 500 mg) or placebo, while the other four groups received two doses of inclisiran (200, 300, or 500 mg) or placebo on day 1 and day 90.

The efficacy of inclisiran was evaluated 6 months after the administration of the first dose. The least-squares mean reduction in LDL cholesterol in the single-dose group ranged from 27.9% to 41.9%. A larger reduction in LDL cholesterol was achieved with two doses of inclisiran (35.5–52.6%). With both dosing regimens, LDL cholesterol reduction was significantly greater compared to placebo, where LDL cholesterol showed an increase of approximately 2% (*p* < 0.001) [24].

The maximum lipid-lowering effect was achieved with the two doses of 300 mg inclisiran (52.6%), similarly to LDL cholesterol reduction with PCSK9 monoclonal antibodies [9]. Almost half of the patients from inclisiran groups (48%) had sustained LDL cholesterol reduction to levels <1.3 mmol/L at day 180. These reductions were followed by the reduction in PCSK9 in inclisiran-treated patients. By the end of the 8 month study period, the levels of LDL cholesterol and PCSK9 remained lower in all patients who received inclisiran. The PCSK9 level was reduced by 56.1%, while the LDL cholesterol level was reduced by 47.2% compared to baseline, which translates into an absolute LDL cholesterol reduction of 1.52 mmol/L [24].

Adverse events were observed at similar rate in inclisiran and placebo groups (76%), mainly mild or moderate in severity [24]. Serious adverse events were recorded in 11% of patients receiving inclisiran and 8% of patients from placebo groups. The most frequent adverse events were the same as in the phase 1 trial [23], with no difference between the study treatments. While patients receiving placebo had no reactions at the site of the injection, this rate was 5% in patients receiving inclisiran, comparable to PCSK9 antibodies [6,26].

Prespecified analysis of the ORION-1 trial explored the effects of inclisiran on atherogenic lipids and lipoproteins, such as non-HDL cholesterol, apolipoprotein B (ApoB), VLDL cholesterol, lipoprotein(a) (Lp(a)), triglycerides, HDL cholesterol, and apolipoprotein A1 (ApoA1) [27]. Significant reductions in non-HDL cholesterol, VLDL cholesterol, and Apo B were observed at day 180 in patients who received a single inclisiran dose. These reductions were augmented by the second dose of inclisiran. The percentage change in non-HDL cholesterol from baseline ranged from −25% to −37% in the single-dose groups and from −32% to −46% in the two-dose groups. The change in VLDL cholesterol showed greater variability, ranging from −15% to −24% with the single-dosing regimen, and reaching approximately −16% with the two-dose regimen. At day 180, ApoB was reduced by −23% to −33% by a single dose of inclisiran, and by −28% to −41% in the two-dose groups. There was a more modest and variable reduction in triglyceride level, where only 300 mg and 500 mg single-dose groups showed a significant reduction (−13% from baseline, and −12% from baseline, respectively), in addition to the 300 mg two-dose group (−14% from baseline). There was a statistically significant increase in HDL cholesterol in patients who received 300 mg of inclisiran as a single dose (+9% from baseline), as well as in all two-dose regimen groups (ranging from +8 to +10%). This was followed by a modest increase in ApoA1, ranging from +3% to 9% in all inclisiran groups. Lastly, Lp(a) reduction over 180 days did not reach statistical significance, with a marked variability in single-dose groups (−14% to −18%) and in two-dose groups (−15% to −26%) [27].

Another prespecified analysis of the ORION-1 trial focused on possible hematological side-effects and immunogenicity of inclisiran [28]. This was the largest safety assessment of siRNA-based therapy so far. Inclisiran at different dosing regimens did not exert side-effects on platelet levels, with no significant change in the number of other leukocytes compared to placebo.

The pleoitropic effects of statins on inflammation, atherosclerosis, and oxidative stress led to the assumption that other lipid-lowering agents, such as PCSK9 inhibitors and inclisiran, may demonstrate similar effects [29]. However, PCSK9 inhibition by inclisiran did not result in a significant reduction in C-reactive protein (CRP) [24,30]. There were no changes in other proinflammatory markers, such as interleukin-6 (IL-6) and tumor necrosis factor-alpha (TNF-α), with any of inclisiran’s explored dosing regimens [28]. However, experimental studies showed that PCSK9 silencing may reduce the levels of vascular inflammation regulators, such as TNF- α, interleukin-1 (IL-1), monocyte chemoattractant protein-1 (MCP-1), Toll-like receptor 4 (TLR4), and nuclear factor kappa B (NF-κB) [31]. In that way, PCSK9 silencing may reduce inflammation in atherosclerotic plaque and stabilize the plaque. Since PCSK9 inhibitors have already demonstrated plaque-stabilizing properties [32,33,34], similar effects may be expected from inclisiran. Other pleiotropic effects of inclisiran, such as the antithrombotic, antibacterial, and antineoplastic effects are yet to be explored.

Prespecified analysis of ORION-1 trial allowed the study of 6068 serum samples for anti-inclisiran antibodies. There was no immunogenicity signal, and administration of inclisiran did not induce any relevant antidrug antibodies [29].

## 5. ORION-1 Trial: 1 Year Follow-Up

A durable effect of siRNA therapy targeting PCSK9 was proven with the 1 year follow-up of patients from the ORION-1 trial [30]. The success of inclisiran was assessed by selecting participants whose LDL cholesterol levels did not return to within 20% of their change from baseline up to day 360. There were 48.3% to 65.0% of such participants in the single-dose group, and between 55.9% and 83.1% from the two-dose group in whom inclisiran demonstrated prolonged effect. Further analysis showed that a single dose of inclisiran resulted in a time-averaged reduction of LDL cholesterol from 29.5% to 38.7%, while the two-dose regimen provided larger effect, with the reduction in LDL cholesterol ranging from 29.9% to 46.4% [30].

The largest number of inclisiran responders during the 1 year follow-up were from a group treated with two doses of 300 mg inclisiran. Moreover, this dosing regimen produced the greatest reduction in LDL cholesterol over 1 year, while inclisiran at 300 mg as a single dose led to a 36.6% reduction in LDL cholesterol level. Additionally, two doses of 300 mg inclisiran given 3 months apart (day 1 and day 90) can provide a 50% reduction in LDL cholesterol lasting at least 6 months after administration of the second dose. Therefore, ORION-1 provided the rationale for the assessment of inclisiran in the following dosing regimen: day 1, day 90, and then twice a year. Moreover, distribution of adverse events was similar in inclisiran and placebo groups, with no new safety signal on day 360 of follow-up [30].

## 6. Four Years of Efficacy and Safety of Inclisiran—ORION-3 Trial

The results of the ORION-3 study were recently published [25]. The ORION-3 trial was an open-label extension study of ORION-1 trial with a 4 year follow-up. Participants who completed the original ORION-1 trial were divided into two groups. The inclisiran only arm consisted of patients that received inclisiran in ORION-1 study (290 of 370). These participants continued with inclisiran regimen—300 mg twice yearly as subcutaneous injections. The second arm, i.e., the switching group, encompassed patients originally allocated to the placebo arm in the ORION-1 trial (92 of 127). These participants transitioned to evolocumab (subcutaneous injection, 140 mg every 2 weeks until day 360). After that, there was a second transition to inclisiran, 300 mg twice yearly until the end of the ORION-3 study [25].

The long-term efficacy of inclisiran was evaluated in the inclisiran only arm until day 210 of the open-label extension period. These patients received inclisiran for a total of 570 days. LDL cholesterol was reduced by 47.5% (95% CI 50.7–44.3) at day 210, with sustained reduction over 1440 days. Therefore, the averaged mean LDL cholesterol reduction in the period of years 1–4 was 44.2% (95% CI: 47.1–41.4), which was an impressive result [25].

The safety profile of inclisiran was comparable to previously published results [24,30]. The most common adverse events in the inclisiran arm were nasopharyngitis (19%) and adverse events at the injection site (14%). However, the transition from evolocumab to inclisiran appeared to be safe, which is valuable information for everyday clinical practice. During the 4 year study period, at least one adverse event at the injection site showed the same frequency in the inclisiran only group (14%, 39 of 284 patients) and in the switching arm (14%, 12 of 87 participants). Treatment-induced serious adverse events had a similar distribution in the inclisiran only and switching arms (37% and 34%, respectively). Although eight deaths were reported during the study period, none of which were treatment-related [25].

Several limitations should be mentioned, such as possible selection bias, since patients who completed the ORION-1 trial voluntarily continued with the extension study. Patients switched to inclisiran, “skipped” the usual dose at day 90, and immediately started with a 6 month dosing regimen. In addition, there was no direct comparison between evolocumab and inclisiran effects on LDL cholesterol levels.

## 7. Patients with Heterozygous Familial Hypercholesterolemia: ORION-9

Patients with heterozygous familial hypercholesterolemia (HeFH) are at risk of premature ASCVD and its early complications [35]. Early diagnosis and rapid initiation of treatment reduces this risk, with many patients with existing ASCVD requiring intensive lipid-lowering therapy. Considering those facts, patients with HeFH were suitable and compelling for further research of inclisiran’s lipid-lowering ability.

ORION-9 was a phase 3 clinical study, which included 482 patients with HeFH with LDL cholesterol level ≥2.6 mmol/L [10]. Patients were randomized to receive inclisiran (300 mg) or placebo as subcutaneous injections on day 1 and 90, followed by two additional doses 6 months apart (day 270 and day 450). The average age of the study population was 56 years, with a baseline LDL cholesterol level of 3.96 mmol/L.

Both primary endpoints were achieved. First, the percent change in LDL cholesterol level at day 510 was reduced with inclisiran therapy (by −39.7%), while patients in the placebo group had an average increase of +8.2% (between-group difference was −47.9%). The second endpoint was the time-averaged percentage change in LDL cholesterol between day 90 and day 540. Similarly, there was a reduction in the inclisiran group (−38.1%), accompanied by an increase in the placebo arm (+6.2%). Similar findings were observed for PCSK9 levels, which was reduced by −60.7% at day 510 with inclisiran and increased by 17.7% with placebo, along with −284.6 µg/L vs. +44.0 µg/L time-averaged differences between day 90 and day 540, respectively [10]. It should be mentioned that inclisiran led to a reduction in Lp(a) by −17.2% from baseline, which is an additive benefit of siRNA-based therapy in patients with HeFH [10].

There was a similar distribution of adverse events in both inclisiran and placebo groups (76.8% and 71.7%, respectively). The majority of these events (92–94%) were mild to moderate in severity. As previously reported, patients receiving inclisiran more frequently reported injection-site reactions (17% vs. 7%), mild in terms of severity [10].

Overall, these findings were similar to results seen in trials of PCSK9 inhibitors in patients with HeFH [18,23,36,37], with the possibility of achieving greater drug adherence with twice-yearly inclisiran administration.

## 8. Patients with ASCVD or ASCVD Risk Equivalent: ORION-10 and ORION-11

Two randomized, placebo-controlled, phase 3 studies further explored the position of inclisiran in primary and secondary cardiovascular prevention [11]. The ORION-10 trial focused on patients with existing ASCVD, while the ORION-11 study included patients with ASCVD or ASCVD risk equivalents (type 2 diabetes, familial hypercholesterolemia, or a 10 year risk of a cardiovascular event of ≥20% as assessed by the Framingham Risk Score for Cardiovascular Disease or equivalent). These studies had matching design, aiming to assess efficacy and safety of inclisiran in patients at high risk for cardiovascular disease. Moreover, all included patients (1561 in ORION-10, and 1671 in ORION-11 trial) had elevated LDL cholesterol level (≥1.8 mmol/L or, in the case of ASCVD risk, a higher threshold of ≥2.6 mmol/L) despite the maximum tolerated statin dose.

The baseline mean value of LDL cholesterol was 2.71 ± 0.99 mmol/L in the ORION-10 and 2.73 ± 1.01 mmol/L in the ORION-11 trial. Each patient during the study period received four subcutaneous injections. Depending on randomization, patients received inclisiran (300 mg) or placebo on days 1 and 90 and then 6 months apart (day 270 and day 450). The final study visit and evaluation were on day 540 [11].

Efficacy was assessed by two primary endpoints, similarly to the ORION-9 trial. The first was an average placebo-corrected change in LDL cholesterol level from baseline to day 510, which was −52.3% in ORION-10 and −49.9% in ORION-11. The second endpoint was the time-adjusted percentage change in LDL cholesterol level from baseline after day 90 up to day 540, which was −53.8% and −49.2%, respectively. Prespecified cardiovascular composite endpoints showed a tendency toward lower cardiovascular risk with the administration of inclisiran (7.4–7.8% vs. 10.2–10.3%, respectively) [11].

Adverse events had a similar distribution in the inclisiran and placebo groups (73.5% and 74.8% in the ORION-10 trial, and 82.7% and 81.5% in the ORION-11 trial, respectively). Again, the majority of adverse events were mild to moderate, with injection-site reaction reported more frequently with the administration of inclisiran in both trials. During the 18 month follow-up, serious adverse events were reported at a similar rate with inclisiran and placebo administration in the ORION-10 trial (22.4% vs. 26.3%, respectively) and in the ORION-11 trial (22.3% vs. 22.5%, respectively) [11].

Recently published patient-level analysis combined the results of the ORION-9, -10, and -11 trials in order to explore cardioprotective effect of inclisiran in a total of 3665 patients with HeFH, ASCVD, and ASCVD risk equivalent [38]. The majority of patients (92%) were receiving statins, including 74% on high-intensity statins. However, only 14% of patients were taking ezetimibe. Such a low percentage of ezetimibe prescription was in contradiction to current dyslipidemia guidelines, which recommend adding ezetimibe in patients where the LDL cholesterol goal is not achieved with the maximum tolerated dose of statin [2]. Therefore, the results of this patient-level analysis should be interpreted cautiously. In this publication, the prespecified exploratory endpoint of major adverse cardiovascular events (MACEs) was defined as a composite of cardiac death, cardiac arrest, nonfatal myocardial infarction, and fatal and nonfatal stroke. Additionally explored outcomes were the occurrence of fatal and nonfatal myocardial infarction, as well as fatal and nonfatal stroke.

For 18 months, MACEs were recorded in 303 (8.3%) patients. Patient-level analysis showed that inclisiran reduced the risk of composite MACEs by 26% (OR 0.74, 95% CI 0.58–0.94) compared to placebo. On the other hand, there was no reduction in fatal and nonfatal myocardial infarction (OR 0.80, 95% CI 0.50–1.27) or in fatal and nonfatal stroke (OR 0.86, 95% CI 0.41–1.81) [38]. This was just a prespecified exploratory analysis and estimation of inclisiran’s cardiovascular benefit in high-risk population; larger studies with longer follow-up are needed.

## 9. Inclisiran in Patients with Polyvascular Disease

Combining the results from three phase 3 studies gave the opportunity to explore inclisiran’s effect on subgroups of patients with polyvascular disease [39]. This was a post hoc analysis of 3454 patients from the ORION-9, -10, and -11 trials. Polyvascular disease (PVD) was defined as the presence of at least two of the following conditions: peripheral artery disease, coronary artery disease, or cerebrovascular disease. Patients were categorized into the PVD group (n = 470; 13.6%) and non-PVD group (n = 2984; 86.4%). As previously mentioned, patients from all three included studies were randomized to receive inclisiran (300 mg) or placebo as subcutaneous injection on day 1 and day 90, followed by twice a year.

The lipid-lowering effect of inclisiran was persistent despite the presence or the absence of PVD. The average placebo-corrected LDL cholesterol percentage change from baseline to day 510 was −48.9% in the PVD group and −51.5% in the non-PVD group [39]. In each group, the effect of inclisiran was significantly greater than in the placebo arms (*p* < 0.0001). The safety of inclisiran administration did not differ between patients with PVD and the subgroup without PVD, except for more frequent adverse reactions at the injection site in both inclisiran arms, along with a moderate increase in bronchitis cases in patients with PVD treated with inclisiran.

Since patients with PVD have very high cardiovascular risk, rapid and aggressive treatment of dyslipidemia is mandatory. Therefore, inclisiran might be a valuable therapeutic option for patients with polyvascular disease who do not achieve LDL cholesterol goal with standard lipid-lowering agents.

The potential of inclisiran to reduce adverse cardiovascular events is being explored in the ongoing VICTORION-2 Prevent [12] and ORION-4 [13] trials. Each trial has an estimated enrollment of 15,000 participants and long-term follow-up. The VICTORION-2 Prevent study is testing the possibility of inclisiran to reduce the risk of composite CV death, nonfatal myocardial infarction, and nonfatal ischemic stroke in patients with ASCVD and elevated LDL cholesterol levels despite well-tolerated high-intensity statin therapy [12]. The ORION-4 trial is enrolling a similar patient population with existing ASCVD treated with either inclisiran (as baseline, 3 months, and then every 6 months) or placebo, with a median follow-up of 5 year for MACEs (coronary heart disease death, myocardial infarction, fatal or nonfatal ischemic stroke, or urgent coronary revascularization) [13].

## 10. Conclusions

A long-term and persistent reduction in LDL cholesterol is the goal of every cholesterol-lowering therapy. However, medication burden and type of treatment regimen may lower therapy adherence and significantly impact the future cardiovascular risk. PCSK9 inhibition by monoclonal antibodies emerged as a novel and powerful treatment approach for lowering circulating PCSK9. Intracellular targeting of PCSK9 with inclisiran exerts beneficial effects on LDL cholesterol and other atherogenic lipids and lipoproteins, beyond those achieved with PCSK9 inhibitors. Twice-yearly administration of inclisiran provides a robust and persistent LDL cholesterol-lowering effect, with a good tolerability profile. Therefore, infrequent dosing of inclisiran may increase therapy adherence, which is particularly important in high-risk patients. Approval of inclisiran as an addon to statin therapy in patients with HeFH or clinical ASCVD who require additional treatment for lowering LDL cholesterol is an important step not just for treating hypercholesterolemia, but also for bringing siRNA-based therapies closer to more common diseases and everyday clinical practice.

## Figures and Tables

**Table 1:** Overview of the major clinical trials testing inclisiran as a cholesterol-lowering agent.

Trial	No. of Patients	Indication	Inclisiran Dosing	Baseline LDL Cholesterol (Mean)	Lowering of LDL Cholesterol from Baseline (mean)	Follow Up	Ref.
Phase 1	69	Healthy population	- Single dose (25, 100, 300, 500 or 800 mg) - Two doses (125 mg weekly for 4 doses; 250 mg every other week for 2 doses; 300 or 500 mg monthly for 2 doses, with or without statins)	3.40–4.21 mmol/L	- Single dose (≥100 mg): up to 50.6% - Two doses (all): up to 59.7%	84 days	[23]
ORION-1	501	Elevated LDL-C, with or without ASCVD	- Single dose (200, 300 or 500 mg) - Two doses (200, 300 or 500 mg) on day 1 and 90	3.05–3.59 mmol/L	- Single dose: 27.9–41.9% - Two doses: 35.5–52.6%	6 months	[24]
ORION-3	382	Elevated LDL-C, with or without ASCVD (open-label extension of ORION-1 trial)	- Inclisiran only arm (300 mg twice yearly) - Switching arm (from placebo to evolocumab 140 mg once every 2 weeks, and to inclisiran 300 mg twice yearly)	3.17–3.33 mmol/L	- Inclisiran only arm (year 1–4): −44.2% - Switching arm (year 2–4): −45.3%	4 years	[25]
ORION-9	482	HeFH	Multiple dosing: 300 mg on day 1 and 90, and two doses twice yearly (day 270 and 450)	3.91–4.00	- Baseline to day 510: −39.7% - Time-averaged change from day 90 to 540: −38.1%	18 months	[10]
ORION-10	1561	ASCVD, elevated LDL-C	Inclisiran 300 mg, day 1, day 90, and every 6 months	2.69 mmol/L	- Baseline to day 510: −51.3% - Time-averaged change from day 90 to 540: −51.3%	18 months	[11]
ORION-11	1671	ASCVD or ASCVD risk equivalent, elevated LDL-C	Inclisiran 300 mg, day 1, day 90, and every 6 months	2.68–2.77 mmol/L	- Baseline to day 510: −45.8% - Time-averaged change from day 90 to 540: −45.8%	18 months	[11]
VICTORION-2 Prevent *	15,000	ASCVD, elevated LDL-C	Inclisiran 300 mg, day 1, day 90, and every 6 months	/	/	6 years	[12]
ORION-4 *	15,000	ASCVD, elevated LDL-C, age≥ 40 (men) or ≥55 (women)	Inclisiran 300 mg, day 1, day 90, and every 6 months	/	/	5 years	[13]

LDL-C—LDL cholesterol; ASCVD—atherosclerotic cardiovascular disease; HeFH—heterozygous familial hypercholesterolemia; Ref.—reference number. * Ongoing trials.

## Data Availability

Not applicable.
